# Seasonal Shift in Exposure and Accumulation of PFAS and Heavy Metals in High Arctic Reindeer

**DOI:** 10.1021/acs.est.5c11066

**Published:** 2026-01-20

**Authors:** Malin Andersson Stavridis, Tove Petersson, Görkem Deniz Kendir, Shannen Sait, Øyvind Mikkelsen, Vebjørn Veiberg, Tomasz Maciej Ciesielski, Bjørn Munro Jenssen

**Affiliations:** † Department of Arctic Technology, 5108 University Centre in Svalbard (UNIS), PO Box 156 Longyearbyen N-9171, Norway; ‡ Department of Biology, Norwegian University of Science and Technology (NTNU), Trondheim NO-7491, Norway; § Department of Ecoscience, Marine Mammal Research, 1006 Aarhus University, Roskilde DK-4000, Denmark; ∥ Department of Chemistry, Norwegian University of Science and Technology (NTNU), Trondheim NO-7491, Norway; ⊥ Land and Biodiversity, 8019 Norwegian Institute for Nature Research (NINA), Trondheim NO-7485, Norway

**Keywords:** contaminants, metals, PFAS, svalbard reindeer, the Arctic

## Abstract

Seasonal fluctuations in contaminant concentrations are an important, yet often overlooked, aspect of Arctic wildlife monitoring, particularly in species like the Svalbard reindeer (*Rangifer tarandus platyrhynchus*), which exhibit annual fattening and fasting cycles. While seasonal variation in metal concentrations has been observed in this species, little is known about how other contaminant groups, such as per- and poly-fluoroalkyl substances (PFAS), vary with season. In the present study, we report concentrations of total mercury (THg), cadmium (Cd), lead (Pb), and 13 PFAS in the liver and muscle of Svalbard reindeer culled in August 2022 and October 2023. Seasonal differences were observed, with higher concentrations of THg, Cd, and several PFAS in October, likely reflecting an extended foraging time following the winter fasting. The PFAS profiles, dominated by PFOS and long-chain PFCAs, primarily suggest exposure to long-range transported contaminants rather than local sources, highlighting that even remote terrestrial wildlife is affected by emissions from industrialized regions. While Cd and Pb concentrations have decreased compared to levels reported in the 1980s, PFAS concentrations have increased over the past decade. These findings underscore the need for continued monitoring of Arctic terrestrial wildlife, particularly in the context of environmental change and remobilisation of legacy contaminants.

## Graphical abstract

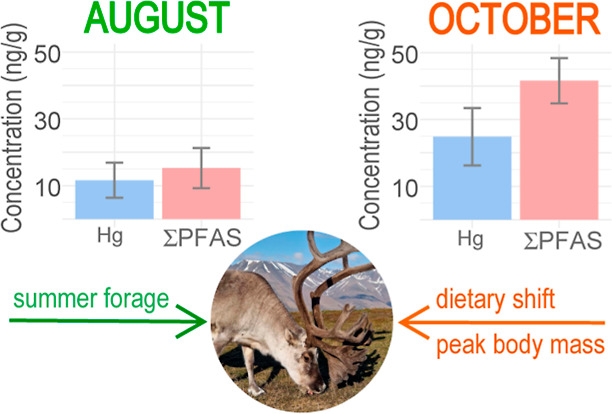

## Introduction

Arctic wildlife is increasingly exposed to a complex mixture of environmental contaminants, often referred to as a chemical cocktail.^1,2^ This cocktail includes toxic metals such as mercury (Hg), cadmium (Cd), and lead (Pb), and per- and poly-fluoroalkyl substances (PFAS), which are all known to cause adverse health effects, such as neurotoxicity, nephrotoxicity, and endocrine disruption.^3,4^

Most contaminants found in the Arctic originate from industrial regions at lower latitudes and are transported northward through long-range atmospheric and oceanic transport.^5–7^ Over time, many of these pollutants have accumulated in the Arctic cryosphere. As the region warms, thawing glaciers, snow, and permafrost may remobilise legacy contaminants, increasing the risk of exposure to Arctic biota.^8,9^ While marine mammals have been the primary focus of Arctic contaminant research, far less is known about terrestrial species, which may face increasing exposure as pollutants re-enter the environment and become available to biota again, for instance, via vegetational uptake,^10^ and subsequent ingestion by herbivores.^11^

The Svalbard reindeer (*Rangifer tarandus platyrhynchus*) is the largest terrestrial herbivore in Svalbard and plays a key ecological role in the local Arctic terrestrial food web.^12^ However, contaminant data for this species remain limited. Borch-Iohnsen et al.^13^ reported strong seasonal variation in hepatic and renal concentrations of toxic metals in Svalbard reindeer culled at the end of the 1980s. Notably, Cd levels were lower in reindeer culled at the end of summer (July/August) compared to autumn (October), while Pb concentrations showed the opposite trend, being higher in late summer and lower in autumn. These shifts were linked to seasonal changes in diet, which reflect variations in exposure to metals throughout the year. In a more recent study, Andersson Stavridis et al.^14^ reported lower Cd and Pb levels in Svalbard reindeer now, compared to four decades ago,^13^ likely due to international emission regulations. In terms of organic contaminants, the study by Roos et al.^15^ remains the only published study on PFAS in Svalbard reindeer. They found that Svalbard reindeer had the lowest PFAS concentrations among the circumarctic *Rangifer* subspecies. However, given the small sample size (*n* = 7) and single time point (2010), it remains unclear whether PFAS concentrations may have changed over the past decade, or whether they, like metals, also follow seasonal patterns.

In this study, we characterize the contaminant profile of Svalbard reindeer liver and muscle tissue collected in August 2022 and October 2023. We quantified concentrations of toxic metals (i.e., Hg, Cd, Pb) and 13 PFAS compounds, including perfluorocarboxylic acids (PFCAs), perfluorosulfonic acids (PFSAs), precursor PFAS, and emerging PFAS. By sampling individuals from the same geographic area across two seasons, we provide new insight into the temporal dynamics of contaminant exposure in Arctic herbivores living in a rapidly changing Arctic environment, where prolonged vegetation growth due to climate warming and pollutant remobilisation may alter their exposure.

## Materials and Methods

### Ethics

All animal sampling was conducted in accordance with Norwegian regulations for the use of wildlife in research. The tissue samples used in this study were obtained from female Svalbard reindeer culled as part of a transdisciplinary international research project,^14^ under permission granted by the Governor of Svalbard, who serves as the responsible licensing authority for wildlife research in Svalbard (permit reference numbers 21/03815-6 and 21/03815-13). No animals were killed specifically for the purpose of this study.

### Samples

The culling campaigns took place in early August 2022 and late October 2023 around Reindalen, Svalbard (77°90′–78°10′ N, 15°30′–16°00′ E). The reindeer were shot through the heart and lung region with regular hunting ammunition and expanding bullets (using bullet types 6.5 × 55 Federal Fusion and 30-06 Lapua Naturalis). All culled reindeer appeared to be in good health, with no visible signs of disease or injury. The age of the reindeer ranged from one to 11 years old, with age being determined using tooth eruption patterns and cementum annuli analysis.^16^ Reproductive maturity was assessed based on the presence of a calf at the time of culling and visible milk production. None of the reindeer culled in August were accompanied by calves, whereas six females culled in October had calves by their side.

In 2022, liver (*n* = 12) and medial thigh muscle (*n* = 12) were sampled within 30 min post-mortem. In 2023, a complete necropsy of the reindeer was performed within 6–12 h post-mortem, during which both liver (*n* = 18) and medial thigh muscle (*n* = 18) samples were collected. To prevent cross-contamination, all samples were collected using clean scalpel blades (Swann-Morton, Swann-Morton Limited, UK) while wearing nitrile gloves. Given the use of lead-containing bullets, samples were exclusively collected from individuals where the entry wound was confined to the heart and lung region, ensuring distance from the tissues of interest. Furthermore, the outer layers (a few mm) of all tissue samples were removed to reduce external contamination of the samples. The samples were stored in polyethylene zip-lock bags (VWR, PA, USA) at −80 °C until further processing.

All samples were freeze–dried (using a FreeZone Benchtop Freeze–Dryer, Labconco, MO, USA) for 72 h at −50 °C and 0.04 mbar. The samples were then placed in clean zip-lock bags before being homogenized using a rubber mallet until a fine powder was obtained.^14^ The weight of the samples was noted (Mettler Toledo, ± 0.01 g) before and after freeze–drying, to determine the moisture content of the samples (an average moisture content of 70.1% in liver and 72.0% in muscle). All subsequent chemical analyses used the freeze–dried sample homogenates. The analyses were conducted at the Department of Chemistry, Norwegian University of Science and Technology (NTNU).

A subset of the collected samples was selected for contaminant analysis, prioritising muscle samples as Svalbard reindeer are recreationally hunted and consumed by locals.^17^ In addition, liver samples representing a range of ages from both culling years were included in the contaminant analysis. Figure 1 illustrates the individual PFAS profiles, indicating which samples were analyzed (colored bars) and which were omitted from the PFAS analysis (white bars). For further details on the culling and sampling sizes, see Table S1.

**Figure 1:**
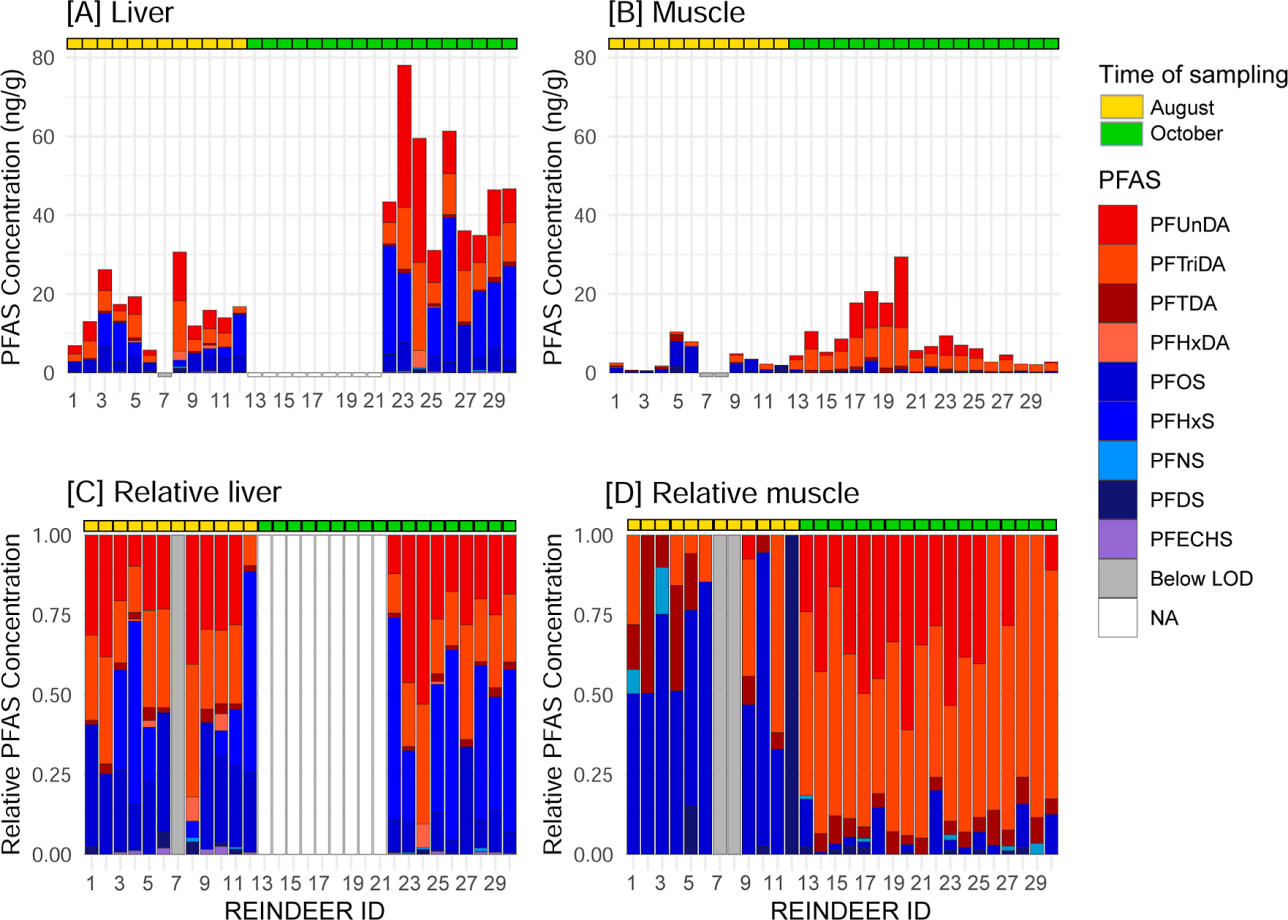
PFCA and PFSA concentrations (ww) in Svalbard reindeer liver [A] and muscle [B], and their relative abundance in liver [C] and muscle [D]. Each bar represents the PFAS fingerprint of a single individual. PFCAs are shown in red and orange, and PFSAs in blue and purple. Light gray bars indicate individuals with PFAS concentrations below the limit of detection (LOD), while white bars (NA) represent hepatic samples that were not analyzed for PFAS. Individuals culled in August are marked in yellow, and those from October are marked in green. Long-range versus local sources of PFAS.

### Mercury Analysis

Liver (*n* = 21) and muscle (*n* = 30) samples were analyzed for total Hg (THg) using a direct mercury analyzer (DMA-80 evo, Milestone, Italy). The analysis followed the US EPA method 7473 with Hg calibration standards, certified reference materials (CRMs), and blanks to ensure analytical accuracy (see Materials and Methods in Supporting Information).

### Elemental Analysis

For analysis of Cd, Pb, and Se, liver (*n* = 21) and muscle (*n* = 20) samples were digested in ultrapure nitric acid (purified from HNO_3_, AnalaR NORMAPUR, VWR) in a sub-boiling distillation system (Milestone, SubPur, Sorisole, BG, Italy) using a high-pressure microwave system (Milestone Ultraclave, EMLS, Leutkirch, Germany) for 150 min. CRMs and blanks were digested following the same protocol as the tissue samples and included in the analysis to ensure analytical accuracy. The elemental concentrations were quantified in the samples, CRMs, and blanks using an 8800 Triple Quadrupole inductively coupled plasma mass spectrometry (ICP-MS) system (Agilent Technologies, USA) equipped with a prepFAST M5 autosampler (ESI, USA), following the protocol described in Andersson Stavridis et al.^14^ In the present study, we report concentrations of toxic elements cadmium (Cd) and lead (Pb), as well as selenium (Se), due to its protective properties against toxic metals.^18,19^ See Materials and Methods in Supporting Information for further details on the analysis, quality assurance, and quality control.

### PFAS Analysis

Liver (*n* = 21) and muscle (*n* = 30) samples were analyzed for 41 different PFAS compounds grouped into perfluoroalkyl carboxylic acids (PFCAs; PFBA, PFPeA, PFHxA, PFHpA, PFOA, PFNA, PFDA, PFUnDA, PFDoDA, PFTriDA, PFTDA, PFHxDA, and PFOcDA), perfluoroalkyl sulfonic acids (PFSAs; PFBS, PFPeS, PFHxS, PFHpS, PFOS, PFNS, PFDS, PFDoDS, and PFECHS), fluorotelomer sulfonates (FTSs; 4:2 FTS, 6:2 FTS, 8:2 FTS, and 10:2 FTS), sulfonamides and precursor PFAS (FOSAA, MeFOSAA, EtFOSAA, PFOSA, MeFOSA, EtFOSA, MeFOSE, and EtFOSE), and emerging PFAS (GenX, NaDONA, 9Cl-PF3ONS, P37DMOA, SaMPAP and diSAMPAP). The analytical method targeted linear PFAS isomers only. The full names of the PFAS compounds are listed in Table S2. PFAS concentrations were determined using an Xevo TQ-XS triple quadrupole mass spectrometer coupled with an ACQUITY UPLC system (Waters, Milford, MA, USA) following the methods described in Sait et al.^20^ The peak integration was performed manually using MassLynx software (Waters, Milford, MA, USA). A detailed extraction protocol is described under Materials and Methods in Supporting Information

### Data Analysis

All contaminant concentrations are presented as average concentrations in ng/g, unless otherwise stated. Only compounds detected above the limit of detection (LOD) in at least 25% of the samples are reported and included in the statistical analysis. Accordingly, concentrations of THg, Cd, Pb, and 13 PFAS are presented for liver, and THg, Pb, and 10 PFAS for muscle. The detection rates for all compounds are provided in Table S3. For these compounds, average and median concentrations were calculated using values above the LOD, while nondetects were excluded from the calculations.

All statistical analyses were conducted using RStudio version 4.3.0.^21^ The data were analyzed for normality using the Shapiro–Wilk test and for equal variances using Levene’s test. Given the non-normal distribution of the raw data, nonparametric Spearman rank correlations were used to assess the relationship between PFAS and biometrics. Annual variations in contaminant concentrations were analyzed using Mann–Whitney U tests with Benjamin–Hochberg corrections applied to limit false discovery rates. Mann–Whitney U tests were also used to assess potential maternal transfer by comparing contaminant concentrations in females with calves to those without among individuals culled in October, but no significant differences were observed.

Statistical analyses were conducted on dry weight (dw) concentrations (Table S3) to avoid any bias introduced by tissue water content. As such, the significant differences shown in Tables 1, 2 and Figure 2 refer to dw, whereas all concentrations in the article are reported on a wet weight (ww) basis, unless otherwise stated.

**Table 1:** Contaminant Concentrations (ww) in Liver Samples from August 2022 and October 2023^a^

liver concentrations (ng/g ww)									
august 2022						october 2023			
contaminant	mean ± SD	median	min	max	mean ± SD	median	min	max	sig.
THg	11.6 ± 5.27	11.1	5.71	25.6	24.8 ± 8.59	21.5	13.1	37.0	(**)
Se^b^	394 ± 57.0	382	314	496	477 ± 56.6	474	393	575	(*)
Cd	192 ± 84.2	178	113	423	532 ± 186	555	329	927	(**)
Pb	52.5 ± 37.8	43.2	14.2	127	48.7 ± 26.5	42.1	13.8	92.7	
Σ_13_PFAS^c^	18.8 ± 13.0	17.1	5.95	54.1	55.8 ± 15.4	53.8	34.8	78.4	(**)
ΣPFCAs	8.93 ± 6.96	7.64	1.93	27.4	26.7 ± 17.0	22.0	11.2	58.3	(**)
ΣPFSAs	7.24 ± 4.81	6.15	2.56	15.1	21.9 ± 11.2	22.9	1.26	39.3	(**)
PFUnDA	4.47 ± 3.13	4.24	1.34	12.4	14.4 ± 11.3	10.1	5.28	36.1	(**)
PFTriDA	4.17 ± 3.17	3.45	1.59	12.8	11.0 ± 5.45	10.3	5.29	22.3	(**)
PFTDA	0.40 ± 0.22	0.40	0.09	0.80	0.87 ± 0.29	0.84	0.42	1.33	(**)
PFHxDA	0.91 ± 0.97	0.62	0.10	2.28	2.32 ± 2.90	2.32	0.27	4.37	
PFHxS	5.35 ± 4.10	3.28	1.23	10.6	21.6 ± 8.30	17.7	12.5	36.7	(**)
PFOS	3.83 ± 1.32	3.84	2.16	6.60	5.28 ± 3.22	4.05	2.44	12.1	
PFNS	0.19 ± 0.21	0.09	0.04	0.42	0.23 ± 0.16	0.31	0.04	0.39	
PFDS	0.36 ± 0.42	0.20	0.10	1.20	0.30 ± 0.41	0.13	0.05	0.91	
PFECHS	0.19 ± 0.10	0.18	0.11	0.39	0.22 ± 0.06	0.23	0.13	0.30	
FOSAA	2.07 ± 5.44	0.19	0.05	16.5	0.78 ± 0.94	0.39	0.01	2.56	
MeFOSAA	0.08 ± 0.10	0.03	0.01	0.20	0.67 ± 0.26	0.52	0.51	0.97	
EtFOSAA	0.93 ± 1.99	0.21	0.01	5.44	0.68 ± 0.88	0.45	0.04	2.63	
NaDONA	0.44 ± 0.46	0.30	0.08	1.49	6.41 ± 7.63	4.94	0.20	23.6	(*)

a Significant differences are marked as (*) when *p* < 0.05, and (**) when *p* < 0.01.

b Se/Hg molar ratio was on average 99.9 in August and 54.4 in October.

c Σ_13_PFAS includes PFUnDA, PFTriDA, PFTDAA, PFHxDA, PFHxS, PFOS, PFNS, PFDS, PFECHS, FOSAA, MeFOSAA, EtFOSAA, NaDONA.

**Table 2:** Contaminant Concentrations (ww) in Muscle Samples from August 2022 and October 2023^a^

muscle concentrations (ng/g ww)									
august 2022						october 2023			
contaminant	mean ± SD	median	min	max	mean ± SD	median	min	max	sig.
THg	2.43 ± 1.49	2.37	0.73	6.37	1.74 ± 0.76	1.41	0.92	3.19	
Se^b^	192 ± 90.0	156	141	424	180 ± 21.3	183	152	205	
Pb	0.23 ± 0.15	0.30	0.03	0.36	0.28 ± 0.24	0.25	0.03	0.73	
Σ_10_PFAS^c^	8.63 ± 12.3	3.45	0.73	33.9	12.7 ± 8.08	6.97	3.09	29.5	
ΣPFCAs	1.12 ± 0.92	1.06	0.06	2.56	8.54 ± 7.19	5.70	1.86	28.5	(**)
ΣPFSAs	2.92 ± 3.37	1.70	0.35	10.9	0.62 ± 0.73	0.44	0.07	3.04	(*)
PFUnDA	below LOD	17.9	2.68	0.30	4.48 ± 4.63	(**)			
PFTriDA	0.98 ± 0.55	0.93	0.27	1.77	4.37 ± 2.67	3.41	1.68	10.6	(**)
PFTDA	0.49 ± 0.57	0.35	0.06	1.84	0.46 ± 0.31	0.35	0.13	1.27	
PFPeS	0.14 ± 0.07	0.14	0.08	0.19	0.10 ± 0.06	0.07	0.04	0.19	
PFOS	2.47 ± 2.49	1.27	0.35	6.72	0.57 ± 0.79	0.32	0.07	3.04	(**)
PFDS	1.20 ± 0.98	1.55	0.09	1.94	0.14 ± 0.10	0.10	0.05	0.37	
FOSAA	4.33 ± 7.10	0.36	0.03	15.7	0.35 ± 0.53	0.14	0.01	2.20	
MeFOSAA	2.26 ± 1.88	2.34	0.13	4.21	0.14 ± 0.09	0.14	0.04	0.23	
EtFOSAA	1.21 ± 1.84	0.23	0.02	4.16	0.25 ± 0.44	0.07	0.01	1.75	
NaDONA	0.39 ± 0.69	0.08	0.03	1.77	0.18 ± 0.45	0.04	0.01	1.67	

a Significant differences are marked as (*) when *p* < 0.05, and (**) when *p* < 0.01.

b Se/Hg molar ratio was on average 228 in August and 321 in October.

c Σ_10_PFAS includes PFUnDA, PFTriDA, PFTDAA, PFPeS, PFOS, PFDS, FOSAA, MeFOSAA, EtFOSAA, and NaDONA.

**Figure 2:**
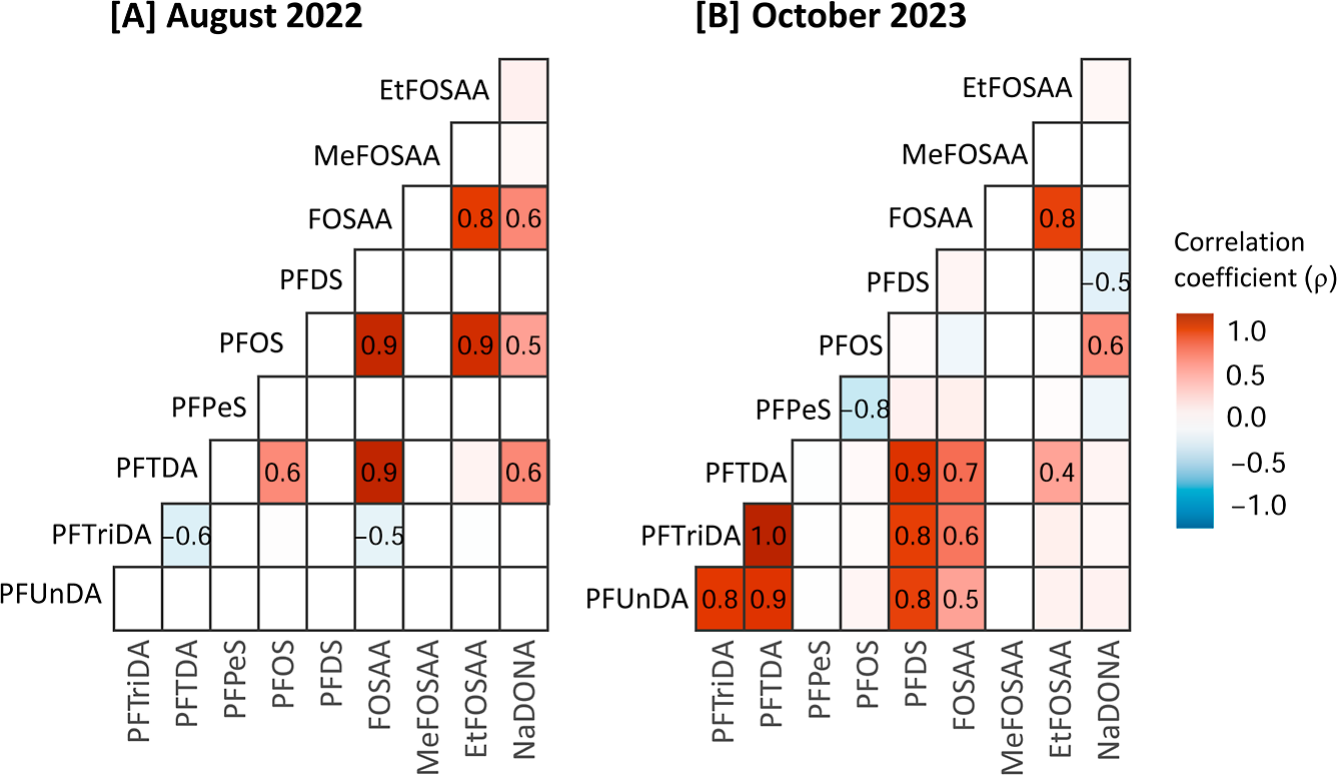
Spearman rank correlation analysis illustrating the relationship between contaminants (dw) in Svalbard reindeer muscle tissue from [A] August 2022 and [B] October 2023. Significant correlations (*p* < 0.05) are visualized with color and ρ-value.

## Results and Discussion

### Seasonal Variation in Svalbard Reindeer Contaminant Levels

In general, we found notable variations in both contaminant levels and profiles between sampling seasons (Tables 1 and 2), and Svalbard reindeer culled in autumn (October) had significantly higher hepatic concentrations of Se, toxic metals (i.e., Hg and Cd), and PFAS, compared to the individuals sampled in late summer (August).

In the present study, average hepatic Hg concentrations for August 2022 and October 2023 were 12 ng/g (ww) and 25 ng/g (ww), respectively. Although previously reported average Hg concentrations for October 2021 (43 ng/g ww) and 2022 (47 ng/g ww) in Svalbard reindeer^14^ were significantly higher than those measured in October 2023, all October data (2021–2023) show consistently higher Hg levels than those measured in August 2022 (*p* = 0.002). A similar pattern between seasons was observed for Cd with significantly higher concentrations in October 2021 (448 ng/g ww)^14^ and October 2023 (532 ng/g ww), compared to the 192 ng/g (ww) measured in August 2022 (*p* = 0.006). Hepatic concentrations of PFAS were also significantly higher in October relative to August, with higher levels of ΣPFAS, ΣPFCAs, and ΣPFSAs (*p* = 0.006, *p* = 0.008, and *p* = 0.015, respectively). Specifically, concentrations of both PFHxS (*p* = 0.008), PFUnDA (*p* = 0.009), PFTriDA (*p* = 0.008), and PFTDA (*p* = 0.009) were severalfold higher in October relative to August.

Similarly to liver, average levels of ΣPFCAs in muscle (Table 2) were significantly higher in October (8.54 ng/g ww) relative to August (1.12 ng/g ww; *p* = 0.002). Conversely, muscle PFOS concentrations displayed an opposite behavior, peaking in August, resulting in a significantly higher ΣPFSAs in 2022 (*p* = 0.023).

Seasonal effects on tissue accumulation of metal concentrations in Svalbard reindeer were previously documented by Borch-Iohnsen et al.,^13^ who suggest that dietary changes influenced by seasonal availability have a significant effect on metal exposure. Seasonal fluctuations in contaminant concentrations are also documented in other Arctic mammals that undergo annual shifts in diet and cycles of fattening and fasting, such as polar bears (*Ursus maritimus*).^22,23^

The Svalbard reindeer undergoes significant seasonal fluctuations in weight, with a peak in body mass (∼70 kg) in October, followed by a decline to approximately 50 kg by April, due to limited winter forage.^24^ This is consistent with the individuals used in the present study, where body mass was slightly lower in August compared to peak mass in October (65 ± 9 kg and 72 ± 9 kg, respectively; Table S1). Given that ingestion of vegetation is the primary exposure route for contaminants in Svalbard reindeer, both the duration of foraging and seasonal shifts in diet are likely to influence their contaminant burden. Reindeer culled in October have had two additional months of foraging compared to those culled in August. We suggest that this extended feeding period may increase cumulative exposure to contaminants present in their diet, or that shifts in diet due to plant phenology and senescence may result in a larger dietary proportion of plant species or parts that contain higher levels of THg, Cd, and ΣPFCAs.

In addition to two additional months of foraging in October, there is a seasonal shift in the types of vegetation consumed from late summer to autumn. While reindeer generally prioritise forage quantity over quality,^25^ changes in plant availability and nutritional content throughout the growing season likely influence dietary composition. Eikeland^26^ reported that certain plant species were frequently grazed in both late summer and autumn, including willow (*Salix polaris*) and grass (*Poa arctica*), while others, such as the flower *Eriophorum scheuchzeri*, were primarily consumed in summer, and the herb *Saxifraga oppositifolia* mainly in autumn. These patterns suggest a shift in grazing preference that may reflect biological changes in nutritional needs or the availability of different types of vegetation as the season progresses. Such dietary shifts are relevant for contaminant exposure, as the concentration of contaminants may vary widely among different species of plants. For example, average ΣPFAS concentrations are higher in vascular vegetation such as *Dryas octopetala* (*x̅* = 8.39 ng/g dw) and *S. polaris* (*x̅* = 2.37 ng/g dw) than in bryophytes (*x̅* = 1.03 ng/g dw).^10^ Likewise, *S. polaris* is known to accumulate Cd, resulting in it having concentrations several times higher than those reported in other vascular plant species.^27^ In contrast, Hg accumulation is more efficient in bryophytes, compared to vascular vegetation.^28,29^ Contaminant burdens in other Arctic cervids have been linked to dietary shifts, with proportions of bryophytes and willows such as *Salix* spp. being important drivers of Hg and Cd exposure.^30^ Consequently, changes in the proportion of different species of vegetation in the diet of the Svalbard reindeer may influence their overall contaminant exposure. In contrast to many other reindeer and caribou across the Arctic, the Svalbard reindeer are nonmigratory, with a home range limited to a few square kilometres.^31,32^ As such, this overall increase in contaminant concentrations between August andOctober is more likely related to a dietary shift, rather than seasonal spatial changes in grazing grounds.

### Tissue Distribution of Contaminants

In addition to differences between seasons, contaminant concentrations also varied significantly between tissues. Overall, liver exhibited the highest concentrations of toxic metals (i.e., Hg, Cd, and Pb), ΣPFAS, as well as the essential element Se (Tables 1 and 2). Notably, Cd concentrations in all muscle samples were below LOD. Despite the large variation in total hepatic PFCA and PFSA concentrations, the PFAS profile in the liver remained relatively homogeneous across both sampling occasions, with PFOS and the long-chained PFCAs PFUnDA and PFTriDA dominating the composition (Figure 1A,C). In contrast, the PFAS profile in muscle was more heterogeneous, with a clear shift between August and October. While PFOS dominated in August, PFUnDA and PFTriDA were most abundant in October (Figure 1B,D).

The differences in contaminant concentrations and profiles between liver and muscle likely reflect several physiological and chemical factors. The overall higher contaminant concentrations in the liver are expected, given that it functions as a major detoxification organ. This may also explain the higher Se concentrations in the liver compared to muscle, given its role in mitigating metal toxicity through the formation of nontoxic complexes,^33–35^ especially in the liver. While Se concentrations in muscle remained stable between late summer and autumn (Table 2), hepatic Se was significantly higher in October than in August (Table 1), possibly due to an increased dietary intake or an enhanced uptake in response to the higher contaminant burdens. Although hepatic concentrations of both Se and Hg increased in October compared to August, the Se/Hg molar ratio was lower in October (54.4) compared to August (99.9), indicating a greater seasonal hepatic accumulation of Hg relative to Se. However, this does not indicate a risk of Se limitations, as a 1:1 molar ratio generally is considered sufficient to mitigate Hg toxicity,^19^ which is far surpassed in both liver and muscle regardless of season.

Higher PFAS concentrations have been reported in the liver compared to muscle in a range of wildlife, including seabirds,^36^ freshwater fish,^37^ and polar bears.^38^ Different mechanisms may influence the distribution of PFAS across tissues, such as variations in chain length and functional groups. Chemical properties, such as lipophilicity, play an important role in tissue distribution, as long-chain PFCAs, with higher logK_o_w values, tend to accumulate in lipid-rich tissues, such as the liver.^39^ In addition to chain length, the functional group of a PFAS influences its distribution in the body by affecting its binding affinity to specific proteins, resulting in tissue-specific accumulation.^38,40^ Additionally, the PFAS half-lives may also be tissue-specific. For example, Drew et al.^41^ found that PFOS has a significantly longer half-life in the liver (116 days) compared to muscle (77 days) in cattle, suggesting that muscle tissue may reflect more recent exposure, while liver reflects longer-term accumulation. Muscle tissue may also be more prone to PFAS remobilisation during periods of energy demand. Trondrud et al.^42^ found that 13–22% of the total energy reserve of Svalbard reindeer is stored in protein. It is, therefore, possible that muscle may be utilized during the annual fasting in spring, resulting in PFAS stored in muscle being remobilised and potentially excreted. Taken together, these mechanisms may help explain the temporal stability observed in the liver PFAS profiles, compared to the more dynamic shifts reported in muscle.

The seasonal shift in the PFAS profile of muscle suggests changes in contaminant exposure between the two sampling seasons (Figure 2). Muscle samples from August were mainly characterized by a strong positive relationship between PFOS and its precursors, FOSAA (ρ = 0.93, *p* = 0.003) and EtFOSAA (ρ = 0.83, *p* = 0.007), whereas samples from October showed stronger intercorrelations among PFDS and long-chain PFCAs (PFUnDA, PFTriDA, and PFTDA; ρ = 0.83–0.96, p < 0.001). Although the precursors FOSAA and EtFOSAA remained strongly correlated in October (ρ = 0.81, p < 0.001), their relationships with PFOS were insignificant. Instead, FOSAA was significantly correlated with PFUnDA, PFTriDA, and PFTDA (ρ = 0.63–0.66, *p* ≤ 0.005). PFOS concentrations were also lower in October (Table 2).

Although the muscle PFAS profiles differed between August and October, both likely reflect exposure dominated by long-range atmospheric transport. However, the specific sources or processes contributing to this long-range input remain uncertain. Volatile precursors such as FOSAA, EtFOSAA, and MeFOSAA reach remote regions like the Arctic via atmospheric transport, and then undergo degradation into both PFSAs (e.g., PFOS) and PFCAs.^9,43^ Hartz et al.,^8^ for example, detected both PFOS, PFAS precursors, and long-chain PFCAs throughout a glacial ice core from Svalbard, demonstrating their continuous deposition following long-range transport. Once deposited, these PFAS may be remobilised into the local environment with meltwater runoff, providing a pathway of exposure to wildlife,^8,44^ such as Svalbard reindeer.

In addition to PFOS and its precursors co-occurring in the Svalbard environment, making them available for direct exposure to wildlife, the PFOS in Svalbard reindeer may partly reflect exposure to the precursor compounds alone, as both FOSAA and EtFOSAA are well-documented to metabolize into PFOS in mammals,^45^ and exposure to these compounds has been suggested to contribute to the accumulation of PFOS in other Svalbard wildlife.^46^ This explanation is supported by the strong intercorrelation between PFOS, FOSAA, and EtFOSAA in August 2022, when concentrations of both PFOS and its precursors were highest. Meanwhile, the absence of a significant relationship in October 2023, when concentrations of both PFOS and its precursors were lower, further suggests that precursor exposure and subsequent biotransformation may have played a role in the PFAS composition of the Svalbard reindeer in August.

It should be noted that the significantly higher muscle concentrations of PFOS observed in August 2022 compared to October 2023 (Table 2) may, to some extent, also be attributed to local emissions. Local sources of PFAS, such as firefighting foam at Longyearbyen Airport^46^ or historical use at the former mining site of Svea,^47^ could contribute to PFOS exposure through local transport to Reindalen. Specifically, the large-scale soil remediation project in Svea between 2020 and 2022 ^48^ may have remobilised PFAS-contaminated particles, which may have spread with the wind to nearby locations, temporarily increasing exposure to PFAS. However, while local sources may play a role in exposure, the relationship in muscle between PFOS and its precursors in August 2022 and the strong intercorrelation between long-chain PFCAs and FOSAA in October 2023 (Figure 2), suggests that long-range transport, rather than local emissions, is the dominant influence on the PFAS profiles in the Svalbard reindeer of the present study. While both profiles (i.e., in August and October) suggest that the PFAS origin is dominated by long-range transport, the shift between the sampling times remains unexplained but likely reflects a combination of changes in atmospheric transport patterns, degradation processes, and seasonal melting of the cryosphere, all of which can influence the availability and composition of PFAS in Arctic ecosystems.

### The Svalbard Reindeer Compared with Other *Rangifer* Subspecies

In line with recent findings,^14^ the concentrations of both Hg and Pb in the present study are among the lowest reported for other *Rangifer* subspecies across the Arctic^49–53^ ; (Figures S1 and S2), while Cd concentrations appear similar across circumpolar subspecies of *Rangifer* ^49,51,52^ ; (Figure S3).

In comparison, few studies have quantified PFAS concentrations in Arctic terrestrial wildlife.^54^ Among those studying reindeer, considerable regional and population differences in both PFAS levels and homologue patterns have been observed. For example, while PFNA and PFDA concentrations were below the limit of detection in both liver and muscle in the present study, these compounds were among the most abundant in both reindeer from Greenland (5.77–17.2 ng/g ww)^15^ and caribou from Canada (1.43–2.85 ng/g ww).^55^ Roos et al.^15^ documented significant differences in hepatic PFAS concentrations across reindeer populations, with PFUnDA levels ranging from 2.66–4.04 ng/g (ww) in Bathurst (Canada), 7.63–22.4 ng/g (ww) in Akia-Maniitsoq (Greenland), and 0.02–0.37 ng/g (ww) in Svalbard. Notably, there were large variations in hepatic PFAS profiles within the same regions, too, with reindeer from Akia–Maniitsoq having PFUnDA concentrations (7.63–22.4 ng/g ww) nearly 5-fold those of the reindeer in the neighboring region of Kangerlussuaq–Sisimiut (1.38–4.01 ng/g ww).^15^ These differences were attributed to factors such as proximity to military installations and airports, local differences in vegetation and climate, and exposure to sea spray aerosols. Hepatic PFUnDA concentrations in the present study (1.34–12.4 ng/g ww) are comparable to those in the caribou from Canada and Greenland, but far higher than what was previously reported in Svalbard reindeer.^15^

Roos et al.^15^ is the only published study reporting PFAS concentrations in Svalbard reindeer. Although the sample size in their study was limited (*n* = 7), the reported concentrations remain interesting to discuss as their samples were collected during the hunting season of 2010, providing a temporal data point to compare our results with. In general, their findings revealed relatively low hepatic PFAS concentrations, and a homologue pattern dominated by PFNA and PFOS. In contrast, our study reports higher overall hepatic concentrations of both PFSAs and PFCAs, along with a shift in the PFAS signature, with long-chain PFCAs such as PFUnDA and PFTriDA dominating instead (Figures S4–S7). These differences may be attributed to spatial variations in sampling in Svalbard or temporal changes in PFAS exposure over the past decade.

Global emissions of PFOS have decreased following the industrial phase-out^56^ and implementation of restrictions on its use and production in 2009.^57^ Yet, PFOS continues to be detected at high concentrations in the tissues of many wildlife species. Recent findings suggest that concentrations in marine predators such as polar bears and ringed seals (*Pusa hispida*) initially decreased following the restriction, but have been increasing again since 2014,^58^ possibly due to enhanced environmental remobilisation of legacy PFAS or dietary shifts. The fact that we observe a similar trend of increasing concentrations of both PFOS and long-chain PFCAs in our study suggests that terrestrial herbivores, such as Svalbard reindeer, may also be affected by these broader environmental changes, affecting their exposure to PFAS.

### Human Consumption of Svalbard Reindeer

Based on the combined intake of PFOA, PFOS, PFNA, and PFHxS (Σ_4_PFAS), which are the four PFAS most commonly found in human blood, a tolerable weekly intake (TWI) of 4.4 ng/kg body weight was established by the European Food Safety Authorities (EFSA) in 2020.^59^ Regarding human consumption of Svalbard reindeer, intake should be approached with caution. According to the present study, Svalbard reindeer in October have an average Σ_4_PFAS of 26.9 ng/g (ww) in liver and 0.57 ng/g (ww) in muscle, respectively. For a 70 kg adult, the TWI corresponds to 308 ng Σ_4_PFAS per week, which would be exceeded by consuming 11.5 g of liver or 540 g of meat. It is worth noting that the PFAS profile of Svalbard reindeer meat and offal not only includes PFOS and PFHxS, but is dominated by long-chain PFCAs, which are not included in the EFSA Σ_4_PFAS calculations, making the comparison between our findings and the TWI less certain. If considering all the PFAS in the ΣPFAS of muscle and liver in the present study, far lower amounts could be consumed without exceeding the set threshold. In addition, although hepatic and muscle concentrations of Hg and Pb remain below toxicity thresholds, hepatic Cd levels in most individuals culled in October exceed the limit of 500 ng/g (ww) set by the European Commission,^60^ which is in line with previous findings by Andersson Stavridis et al. (2025).^14^ Nevertheless, since Svalbard reindeer hunting occurs on a small scale, with each hunter being allocated a quota of one animal per year, the overall contaminant exposure risk is likely low but should still be considered.

High concentrations of PFAS in wild game meat are not limited to Arctic wildlife, and studies from different regions around the world report concentrations of contaminants in game that exceed intake thresholds. For instance, elevated levels of PFAS have been documented in white-tailed deer (*Odocoileus virginianus*) in the midwestern USA,^61^ and in wild boar (*Sus scrofa*) from the Czech Republic,^62^ Poland,^63^ and Germany.^64^ These findings have led to governmental actions, such as hunting restrictions, and highlight the need for further studies on wildlife living close to local PFAS emission sources or in environments such as the Arctic, where legacy pollutants can remobilise in the future.

### Implications

The current study reveals significant seasonal differences in contaminant concentrations, with higher concentrations in Svalbard reindeer culled in October compared to those culled in August. With the current annual hunting period starting in mid-August and ending at the end of September,^65^ our findings suggest that hunters should aim to harvest their licensed animals as early as possible in the allowed period to minimize their dietary exposure to contaminants. The seasonal differences observed in this study also highlight the importance of carefully planning future monitoring programs to ensure that all samples are collected at the same time of year, as consistency is necessary for the results to be comparable across years.

While the concentrations of toxic metals appear to be relatively stable over the past three years, there has been a significant increase in PFAS in Svalbard reindeer over the past decade. The shift in the PFAS composition of the tissues, from a profile dominated by PFOS to one where long-chain PFCAs are equally abundant, may be due to the remobilisation of legacy contaminants from the melting cryosphere as the Arctic warms. This increase in PFAS highlights the need for further monitoring to assess the impact of a rapidly changing Arctic on terrestrial wildlife.

## Supplementary Material



## Data Availability

All data used during this study are available in the Mendeley Data repository: https://data.mendeley.com/datasets/fpgsn3j373/1 [DOI:10.17632/fpgsn3j373.1]

## Notes

Supporting Information Table S1 provides supplementary culling and chemical analysis information. Table S2 provides the full name and recovery rate of all analyzed PFAS. Table S3 provides all contaminant concentrations in dry weight (dw). Materials and methods provide detailed descriptions of the analytical methods used to determine Hg, elements Cd, Pb, and Se, and PFAS. The supporting figures (Figures S1–S7) compare the concentrations of Hg, Cd, Pb, PFUnDA, PFTriDA, PFHxS, and PFOS with the concentrations reported in other reindeer and caribou across the Arctic (PDF)
