# Supplementary material for: Seasonal Shift in Exposure and Accumulation of PFAS and Heavy Metals in High Arctic Reindeer
Source: Environ Sci Technol. 2026 Jan 20;60(4):3449–58. doi: 10.1021/acs.est.5c11066 (PMC12874527; doi:10.1021/acs.est.5c11066)
Supplement: Supplementary file 1 [file es5c11066_si_001.pdf]

SUPPORTING INFORMATION

Title: Seasonal Shift in Exposure and Accumulation of PFAS and Heavy Metals in High Arctic Reindeer

Authors: Malin Andersson Stavridis[1,2*], Tove Petersson[1,3], Görkem Deniz Kendir[2,4], Shannen Sait[4], Øyvind Mikkelsen[1,4], Vebjørn Veiberg[5], Tomasz Maciej Ciesielski[1] & Bjørn Munro Jenssen[1,2*].

Affiliations:
1. Department of Arctic Technology, University Centre in Svalbard (UNIS), PO Box 156 N-9171, Longyearbyen, Norway.
2. Department of Biology, Norwegian University of Science and Technology (NTNU), NO-7491, Trondheim, Norway.
3. Department of Ecoscience, Marine Mammal Research, Aarhus University, DK-4000, Roskilde, Denmark.
4. Department of Chemistry, Norwegian University of Science and Technology (NTNU), NO-7491, Trondheim, Norway.
5. Land and Biodiversity, Norwegian Institute for Nature Research (NINA), NO-7485 Trondheim, Norway.

*corresponding author(s): Malin Andersson Stavridis: malins@unis.no & Bjørn Munro Jenssen: bjorn.munro.jenssen@ntnu.no.

SUPPORTING INFORMATION CONTAINS

18 pages: S1-S18

Tables S1-S3

Materials and Methods supplementary (with further details on chemical analysis)

Figures S1-S7

TABLE S1. Supplementary culling and analysis information.

					Samples analysed for contaminants					
					Hg		Elements		PFAS	
**ID**	**Culling time**	**Age**	**Weight**	**Location**	**Liver**	**Muscle**	**Liver**	**Muscle**	**Liver**	**Muscle**
1	Aug-2022	5	78	Reindalen	×	×	×	×	×	×
2	Aug-2022	4	65	Reindalen	×	×	×	×	×	×
3	Aug-2022	2	62	Semmeldalen	×	×	×	×	×	×
4	Aug-2022	10	73	Semmeldalen	×	×	×	×	×	×
5	Aug-2022	2	56.5	Reindalen	×	×	×	×	×	×
6	Aug-2022	9	70.5	Reindalen	×	×	×	×	×	×
7	Aug-2022	8	70	Reindalen	×	×	×	×	×	×
8	Aug-2022	9	67	Semmeldalen	×	×	×	×	×	×
9	Aug-2022	8	71	Kalvdalen	×	×	×	×	×	×
10	Aug-2022	2	58	Kalvdalen	×	×	×	×	×	×
11	Aug-2022	1	44	Kalvdalen	×	×	×	×	×	×
12	Aug-2022	3	60	Kalvdalen	×	×	×	×	×	×
13	Oct-2023	8	71	Colesdalen		×		×	×	×
14	Oct-2023	11	66	Colesdalen		×		×		×
15	Oct-2023	7	71	Colesdalen		×		×		×
16	Oct-2023	7	80	Colesdalen		×			×	×
17	Oct-2023	7	76	Colesdalen		×		×		×
18	Oct-2023	9	68.5	Colesdalen		×		×		×
19	Oct-2023	5	78	Colesdalen		×				×
20	Oct-2023	6	69	Reindalen		×		×		×
21	Oct-2023	7	73	Reindalen		×		×	×	×
22	Oct-2023	10	67	Reindalen	×	×	×	×	×	×
23	Oct-2023	10	75	Colesdalen	×	×	×		×	×
24	Oct-2023	7	73.5	Colesdalen	×	×	×			×
25	Oct-2023	8	73.5	Colesdalen	×	×	×			×
26	Oct-2023	4	67	Reindalen	×	×	×		×	×
27	Oct-2023	7	74.5	Reindalen	×	×	×			×
28	Oct-2023	6	72	Colesdalen	×	×	×		×	×
29	Oct-2023	4	70.5	Colesdalen	×	×	×		×	×
30	Oct-2023	6	78	Reindalen	×	×	×		×	×
				n=	21	30	21	20	21	30

TABLE S2. Full name, abbreviation, and recovery rate of all PFAS analysed in the study. Grouped by functional group and chemical structure.

	Full name	Abbreviation	Recovery (%)
PFCAs	Perfluorobutanoic acid	PFBA	100
	Perfluoropentanoic acid	PFPeA	103
	Perfluorohexanoic acid	PFHxA	100
	Perfluoroheptanoic acid	PFHpA	101
	7H-dodecafluoroheptanoic acid	PFHeA	63
	Perfluorooctanoic acid	PFOA	100
	Perfluorononanoic acid	PFNA	107
	Perfluorodecanoic acid	PFDA	105
	Perfluoroundecanoic acid	PFUnDA	55
	Perfluorododecanoic acid	PFDoDA	55
	Perfluorotridecanoic acid	PFTriDA	45
	Perfluorotetradecanoic acid	PFTDA	58
	Perfluoro-n-hexadecanoic acid	PFHxDA	91
	Perfluorooctadecanoic acid	PFOcDA	135
PFSAs	Perfluorobutanoic acid sulfonate	PFBS	100
	Perfluoropentane sulfonic acid	PFPeS	61
	Perfluorohexane sulfonic acid	PFHxS	64
	Perfluoro-1-heptanesulfonate	PFHpS	63
	Perfluorooctano sulfonic acid	PFOS	61
	Perfluorononane sulfonic acid	PFNS	63
	Perfluorodecane sulfonic acid	PFDS	61
	Perfluorododecane sulfonic acid	PFDoDS	63
	Perfluoroethylcyclohexane sulfonic acid	PFECHS	63
FTSs	1H,2H-perfluorohexan sulfonate 4:2	4:2 FTS	62
	1H,2H-perfluorooctane sulfonate 6:2	6:2 FTS	66
	1H,2H-perfluorodecan sulfonate 8:2	8:2 FTS	69
	1H,2H-perfluorododecan sulfonate 10:2	10:2 FTS	62
Sulfonamides and precursor PFAS	Perfluoro-1-octanesulfonamidoacetic acid	FOSAA	81
	2-(N-methylperfluoro-1-octansulfonamido)acetic acid	MeFOSAA	97
	N-ethylperfluoro-1-octanesulfonamide acetic acid	EtFOSAA	94
	Perfluorooctane sulfonamide	PFOSA	80
	N-methylperfluoro-1-octanesulfonamide	MeFOSA	69
	Sulfluramid	EtFOSA	74
	N-(2-hydroxyethyl)-N-methylperfluorooctane sulfonamide	MeFOSE	93
	N-ethyl-N-(2-hydroxyethyl)-N-methylperfluorooctane sulfonamide	EtFOSE	16
Emerging PFAS	2,3,3,3-tetrafluoro-2-(1,1,2,2,3,3,3-heptafluoropropoxy)propanoate	GenX	69
	Sodium dodecafluoro-3H-4,8-dioxanonanoate	NaDONA	69
	9-chlorohexadecafluoro-3-oxanonane-1-sulfonate	9Cl-PF3ONS	67
	2-(N-ethylperfluorooctane-1-sulfonamido)ethyl phosphate	SaMPAP	66
	Bis[2-(N-ethylperfluorooctane-1-sulfonamido)ethyl] phosphate	diSAMPAP	0
	Perfluoro-3,7-dimethyloctanoic acid	P37DMOA	66

TABLE S3. Dry weight (dw) concentrations of contaminants in Svalbard reindeer liver and muscle in August 2022 and October 2023. For each contaminant, columns refer to the detection rate, LOD, mean ± SD, median, minimum, and maximum contaminant concentrations.

			Concentration (ng/g dw)								
			August 2022					October 2023			
**Tissue**	**Contaminant**	**%>LOD**	**LOD**	**Mean±SD**	**Median**	**Min**	**Max**	**Mean±SD**	**Median**	**Min**	**Max**
Liver	THg	100	0.0003	40.4±19.0	37.2	20.4	92.1	83.5±28.5	67.9	49.7	130.2
	Se	100	0.003	1370±209	1340	1030	1700	1610±144	1589	1383	1901
	Cd	100	0.14	671±308	622	407	1520	1811±664	1850	1040	3130
	Pb	100	0.003	180±123	151	52.2	414	163±88.3	142	52.5	326
	PFUnDA	91	0.006	15.6±11.2	14.7	4.59	44.4	46.7±40.1	34.6	16.7	122
	PFTriDA	95	0.006	14.5±11.3	12.3	5.73	43.6	37.9±21.2	33.3	16.8	84.9
	PFTDA	86	0.006	1.37±0.73	1.34	0.31	2.69	2.90±0.94	2.83	1.40	4.29
	PFHxDA	29	0.006	3.17±3.49	2.09	0.31	8.18	8.78±11.1	8.78	0.94	16.6
	PFHxS	67	0.006	18.2±14.0	11.0	4.17	38.0	70.9±25.3	59.7	44.0	117
	PFOS	86	0.02	13.2±4.31	13.7	7.40	21.5	17.8±11.5	13.5	7.75	42.8
	PFNS	38	0.006	0.66±0.74	0.34	0.14	1.51	0.80±0.57	1.00	0.13	1.31
	PFDS	48	0.006	1.27±1.50	0.71	0.31	4.30	1.11±1.58	0.41	0.16	3.47
	PFECHS	57	0.006	0.65±0.35	0.57	0.39	1.32	0.47±0.21	0.77	0.44	0.96
	FOSAA	81	0.006	7.42±19.5	0.70	0.17	59.3	2.70±3.32	1.28	0.04	9.01
	MeFOSAA	29	0.006	0.28±0.37	0.11	0.02	0.71	2.38±1.15	1.82	1.62	3.70
	EtFOSAA	67	0.006	3.31±7.14	0.74	0.05	19.5	2.36±3.11	1.47	0.15	9.26
	NaDONA	76	0.006	1.53±1.66	1.02	0.29	5.34	22.3±27.1	16.32	0.70	83.0
Muscle	THg	100	0.0003	8.53±4.96	8.09	2.74	21.5	6.35±3.04	5.19	3.30	12.9
	Se	95	0.003	678±320	582	490	1500	654±108	670	523	831
	Pb	47	0.003	0.83±0.51	0.98	0.10	1.33	1.00±0.88	0.87	0.09	2.77
	PFUnDA	53	0.006	Below the LOD				16.2±16.2	10.1	1.05	61.1

			Concentration (ng/g dw)								
			August 2022					October 2023			
Tissue	Contaminant	%>LOD	LOD	Mean±SD	Median	Min	Max	Mean±SD	Median	Min	Max
	PFTriDA	80	0.006	3.41±1.93	3.19	1.08	6.08	15.8±9.76	12.0	6.01	38.8
	PFTDA	83	0.006	1.68±1.84	1.22	0.22	5.95	1.68±1.13	1.17	0.46	4.68
	PFPeS	27	0.02	0.49±0.24	0.49	0.32	0.67	0.35±0.24	0.25	0.13	0.73
	PFOS	77	0.02	8.47±8.19	4.42	1.23	22.8	2.06±2.93	1.14	0.26	11.5
	PFDS	40	0.006	3.97±3.24	5.03	0.33	6.55	0.50±0.39	0.38	0.18	1.38
	FOSAA	83	0.006	14.3±23.4	1.29	0.12	50.7	1.27±1.95	0.52	0.03	8.09
	MeFOSAA	27	0.006	7.48±6.14	7.93	0.47	13.6	0.50±0.34	0.53	0.13	0.80
	EtFOSAA	77	0.006	4.02±6.11	0.79	0.07	14.1	0.93±1.63	0.27	0.03	6.46
	NaDONA	63	0.006	1.33±2.31	0.28	0.13	5.97	0.65±1.66	0.17	0.05	6.16

MATERIALS AND METHODS SUPPLEMENTARY

THg determination

Before analysis, Hg calibration standards were made by preparing solutions containing 2% hydrochloric acid, ultrapure water (Elga® Purelab Flex 4), and Hg from stock (LabKings B.V.®; 0.01-100 ng) following the instructions provided by Milestone® for ultratrace analysis of Hg. The calibration standards were used to create calibration curves in both cell 0 (R^2=0.999) and cell 1 (R^2=0.998. Both certified reference materials (CRMs) and calibration standards were analysed to ensure the precision of the analysis, with MODAS–3 Herring Tissue (M–3 HerTis, 221±21 µg/kg, recovery ranging between 107-108%) from MODAS, Gdańsk (2015) and ORIENTAL BASMA TOBACCO LEAVES (INC-OBTL-5, 23.2±1.6 µg/kg, recovery ranging between 80-87%) from the Institute of Nuclear Chemistry and Technology, Warszawa (2010) used as CRMs in cell 0 and cell 1, respectively.

Blanks were run at the beginning and end of each analysis day, with blank heights not exceeding 0.003. All samples were run in singletons with periodic triplicates (about every 10 samples). Approximately 40 mg of sample material was analysed, with less mass analysed for samples with higher fat content (22-40 mg). Background absorbance was accounted for by averaging the blank heights from each day and subtracting this from the height of the sample before calculating the final concentration.

Element quantification

Approximately 400 mg of muscle, 300 mg of liver, 300 mg of CRM (1577b Bovine Liver, US Department of Commerce National Institute of Standards and Technology, Gaithersburg, Md 20899), 300 g of ultrapure water were added to polytetrafluoroethylene (PTFE) vials containing 5 ml of 50% (v/v) HNO_3 (purified from HNO3, AnalaR NORMAPUR®, VWR) in a sub-boiling distillation system (Milestone, SubPur, Sorisole, BG, Italy). Once digested using a high-pressure microwave system (Milestone Ultraclave, EMLS, Leutkirch, Germany), the samples were diluted to a final weight of approximately 54g. Concentrations of elements Cd,

Pb, and Se were quantified in the liver and muscle, CRM (Bovine Liver Standard Reference Material 1577b), and blank digests using an 8800 Triple Quadrupole inductively coupled plasma mass spectrometry (ICP-MS) system (Agilent, USA) equipped with a prepFAST M5 autosampler (ESI, USA). The final sample and CRM concentrations were corrected for total dilution volume and blank value concentrations.

The Bovine Liver 1577b certified values were as follows for Cd: 0.5±0.03 µg/g, Pb: 0.129±0.004, and Se: 0.73±0.06. Reference material recovery rates ranged from 79.9-84.3% for Pb, 84.7-106% for Cd, and 90.2-97.3% for Se.

The limits of detection (LOD) were determined as either three times the standard deviation of the blanks or the instrument detection limits based on the highest obtained value. The LOD for Cd was 0.47 µg/kg, for Pb 0.02 µg/kg, and for Se 0.01 µg/kg. All elements were detected above the LOD in the liver samples. In contrast, Cd was not detected above LOD in a majority of the muscle samples and therefore not reported.

PFAS quantification

Quantification of target analytes was achieved using internal standards and matrix-matched calibration curves, which were prepared by spiking target analytes and internal standards into the matrix prior to extraction, as described in Sait et al., (2023). Calibration was performed using a 13-point calibration curve (0.00, 0.01, 0.02, 0.05, 0.10, 0.20, 0.50, 1.00, 2.00, 5.00, 10.0, 20.0, and 50.0 ng/ml) with R2 values ranging from 0.935-0.999. The method LOD was determined using the lowest detected concentration in the calibration curve range, corrected for the dilution factor, with LODs ranging between 0.013-0.152 ng/g ww. Solvent blanks were run every ten samples to monitor contamination and were subtracted from sample measurements to correct final concentrations.

The efficiency of the extraction was determined through absolute recovery rates using the method blanks, matrix-matched, and spiked samples. Out of the 41 targeted PFAS, EtFOSE

and diSAMPAP had recoveries below 45% and were excluded from the analysis. Recovery rates for the remaining PFAS ranged between 45-135%, with RSDs <10%. Several PFAS were not detected above the LOD in at least 25 % the samples and are consequently not reported in the current study. These include PFBA, PFPeA, PFHxA PFHpA, PFOA, PFNA, PFDA, PFDoDA, PFOcDA, PFBS, PFHpS, PFDoDS, 4:2 FTS, 6:2 FTS, 8:2 FTS, 10:2 FTS, PFOSA, MeFOSA, EtFOSA, MeFOSE, EtFOSE, GenX, 9Cl-PF3ONS, P37DMOA, SaMPAP, and diSAMPAP in both liver and muscle samples, PFHxDA, PFHxS, PFNS, PFECHS in muscle samples, and PFPeS in liver samples.

Analytical standards (purity ≥98%) were supplied by Wellington Laboratories Inc. (Ontario, Canada). Internal standards (purity ≥99%) of isotopically labelled perfluorooctanoic acid (PFOA-13C8), perfluorooctanesulfonate sodium salt (PFOS-13C8), and 1H,2H-perfluorooctane sulfonate (6:2) (6:2 FTS-13C2) were supplied by Cambridge Isotope Laboratories, Inc. (Tewksbury, MA, US). Stock solutions of target analyte (200 ppb) and internal standards (1 ppm) were prepared in methanol (MeOH) and stored at -20^{0}C.

Protocol for PFAS extraction

Approximately 100 mg of homogenised and freeze-dried liver and muscle samples were transferred into individual 15 mL polypropylene (PP) test tubes. To these tubes, 3 mL of ethyl acetate, 300 µL of 1.0 M ammonium acetate, and 10 µL of internal standard mix were added. The tubes were vortexed for 10 seconds until fully mixed, then ultrasonicated for 45 minutes. Following centrifugation (4000×g, 10 min) for protein precipitation, the top 3 mL ethyl acetate layer was transferred to a fresh PP tube. An additional 3 mL of ethyl acetate was added to the original sample tube, followed by vortexing, ultrasonication, and centrifugation. The top 3 mL ethyl acetate layer was again transferred to the fresh PP tube. This step was repeated once more, resulting in a total of 9 mL of ethyl acetate extract. Following the last transfer, 1 mL of ultrapure water was added to the extracts.

The new mixtures were centrifuged once more (4000×g, 10 min), and the supernatant of each tube was transferred to fresh PP tubes. The supernatant solutions were evaporated under a gentle stream of nitrogen gas (~2.5 PSI) to near dryness. The dried residue was reconstituted in 500 µL of a 1:1 MeOH-water solution, vortexed for 10s, and transferred to liquid chromatography (LC) autosampler vials. Vials were stored at -20°C until further analysis.

Analysis of PFAS was conducted at the Norwegian University of Science and Technology (NTNU) using ultra-performance liquid chromatography (UPLC) on a Waters Acquity I-Class system (Waters, Milford, USA), coupled to a Xevo TQ-S triple quadrupole mass spectrometer operated in multiple reaction monitoring (MRM) mode. Ionization was performed using a ZSpray electrospray ion source in negative mode (ESI–). Chromatographic separation was achieved using a Kinetex C18 column (30 × 2.1 mm, 1.3 µm, 100 Å; Phenomenex, Denmark) equipped with a matching C18 guard column (10 × 2.1 mm). The column temperature was held at 30 °C.

Mobile phases consisted of (A) water with 2 mM ammonium acetate and (B) methanol. A gradient elution was applied as follows: 20% B for 0.1 min, ramped to 50% at 0.1 min, 70% at 0.6 min, 80% at 0.7 min, 85% at 1.3 min, and 100% at 1.7 min. This was held for 1 min before returning to 20% B over 0.1 min, followed by re-equilibration for 0.4 min. The total run time was 6 minutes, with a flow rate of 0.25 mL/min and an injection volume of 4 µL.

Instrument parameters were optimized as follows: capillary voltage at 2.0 kV, source temperature 150 °C, desolvation temperature 450 °C, cone gas flow 150 L/h, desolvation gas flow 650 L/h, and nebulizer pressure at 6 bar.

Peak integration was carried out manually using MassLynx software. Final concentrations were corrected for sample mass and dilution factor.

SUPPORTING FIGURES

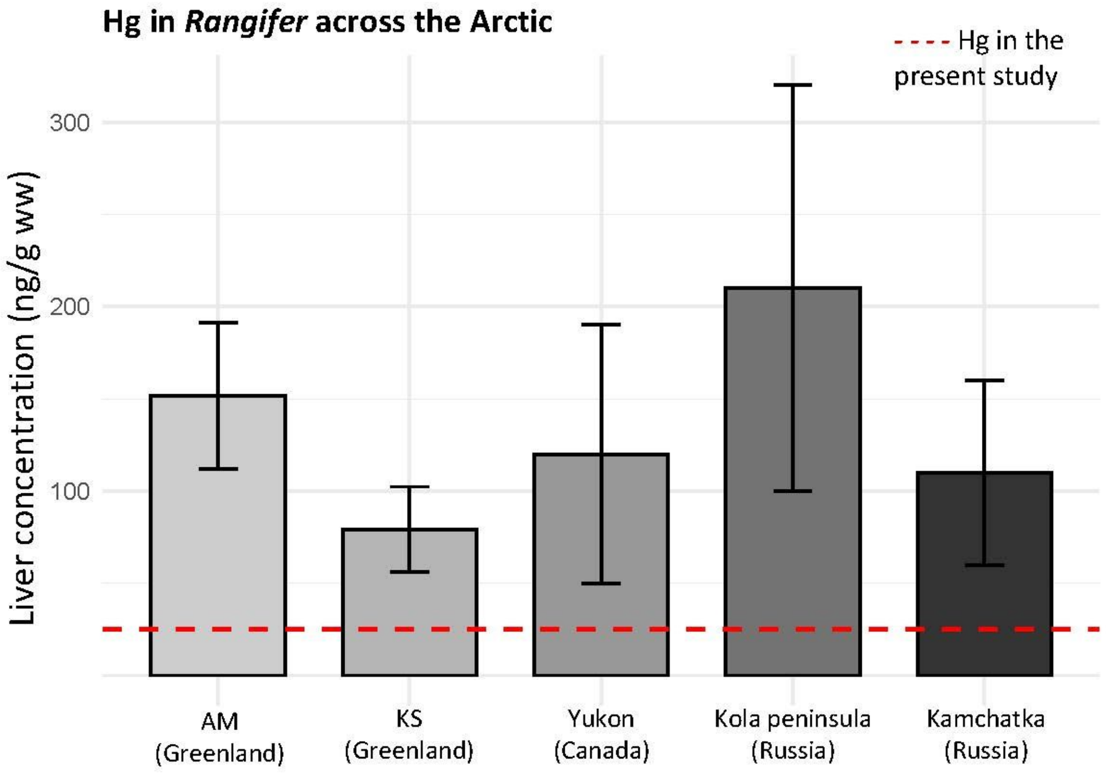

Figure S1. Hepatic Hg concentrations (ww) from various Rangifer subspecies across the Arctic. The dashed red line represents the average Hg concentration reported in individuals culled in October in the present study (25 ng/g ww). Concentrations from Akia-Maniitsoq (AM) and Kangerlussuaq-Sisimiut (KS) are reported in Gamberg et al. (2016), concentrations from Yukon derive from Schuster et al., (2011), and the data from Kola Peninsula and Kamchatka from Makarov et al. (2022).

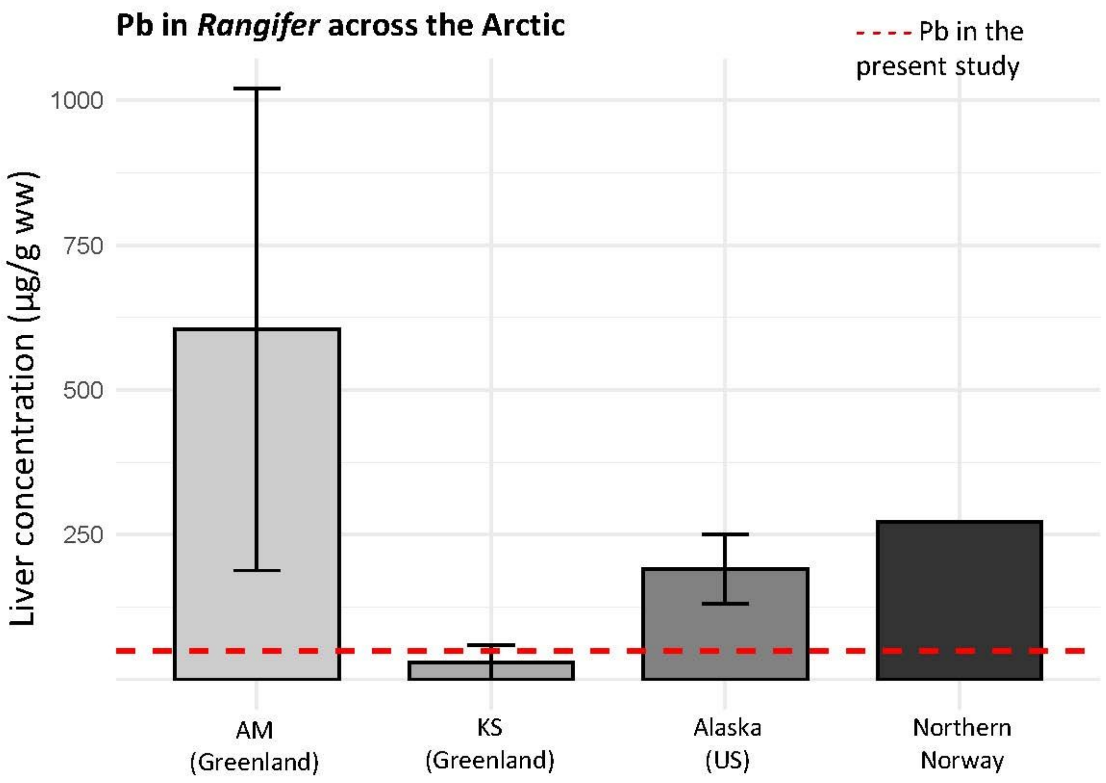

Figure S2. Hepatic Pb concentrations (ww) from various Rangifer subspecies across the Arctic. The dashed red line represents the average Pb concentration reported in individuals culled in October in the present study (49 ng/g ww). Concentrations from Akia-Maniitsoq (AM) and Kangerlussuaq-Sisimiut (KS) are reported in Gamberg et al. (2016), concentrations from Alaska derive from O'Hara et al. (2003), and the data from Northern Norway from Ali Hassan et al. (2012).

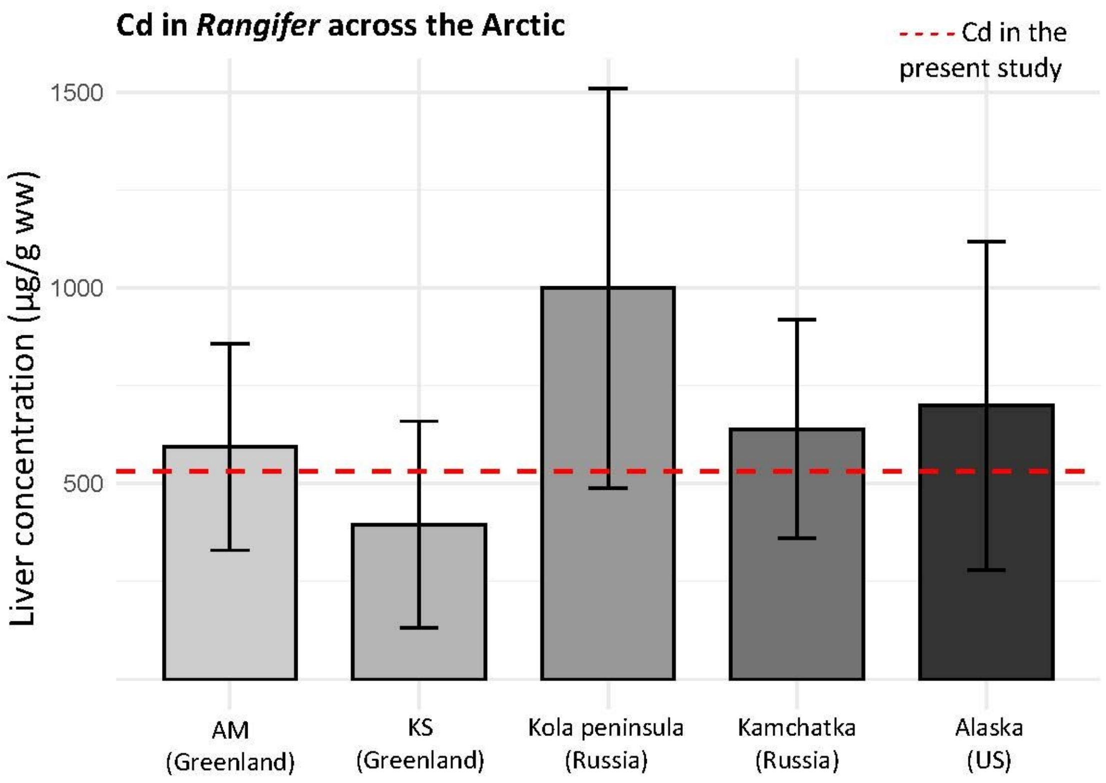

Figure S3. Hepatic Cd concentrations (ww) from various Rangifer subspecies across the Arctic. The dashed red line represents the average Cd concentration reported in individuals culled in October in the present study (532 ng/g ww). Concentrations from Akia-Maniitsoq (AM) and Kangerlussuaq-Sisimiut (KS) are reported in Gamberg et al. (2016), concentrations from Kola peninsula and Kamchatka derive from Makarov et al. (2022) and the data from Alaska from O'Hara et al. (2003).

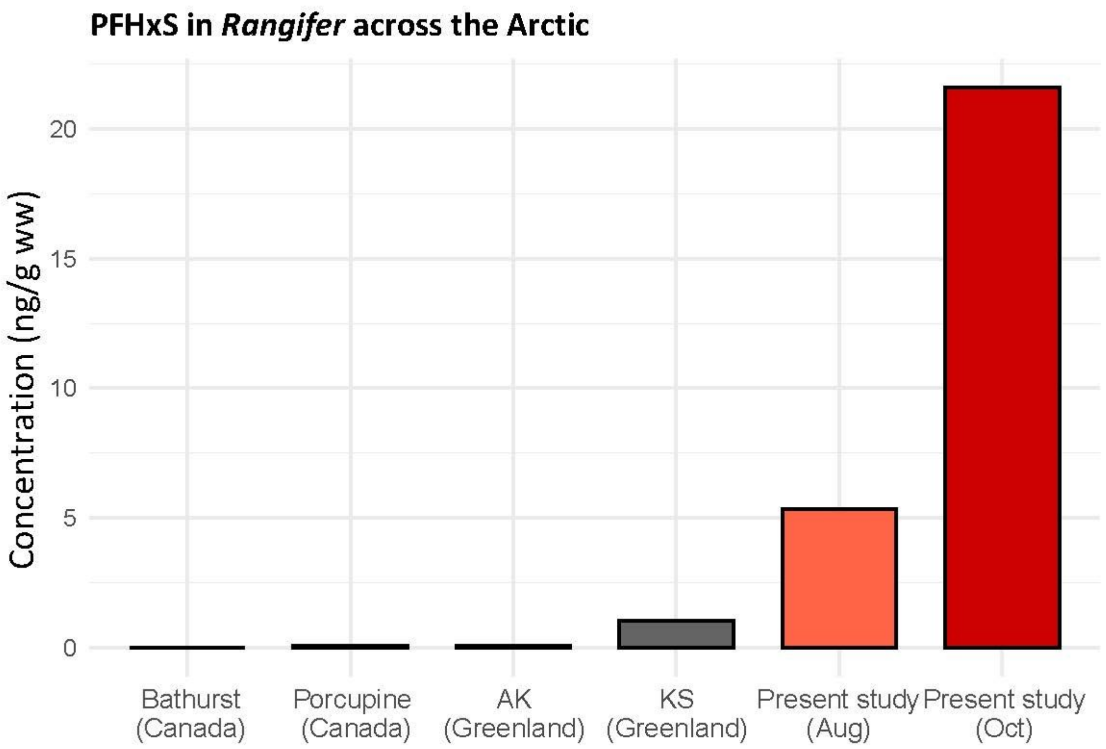

Figure S4. Hepatic PFHxS concentrations (ww) from various Rangifer subspecies across the Arctic. Concentrations from Bathurst, Porcupine, Akia-Maniitsoq (AK) and Kangerlussuaq-Sisimiut (KS) are all reported in Roos et al. (2021) and are here visualised in different shades of grey. Average concentrations from both animals culled in August and October in the present study are visualised in shades of red.

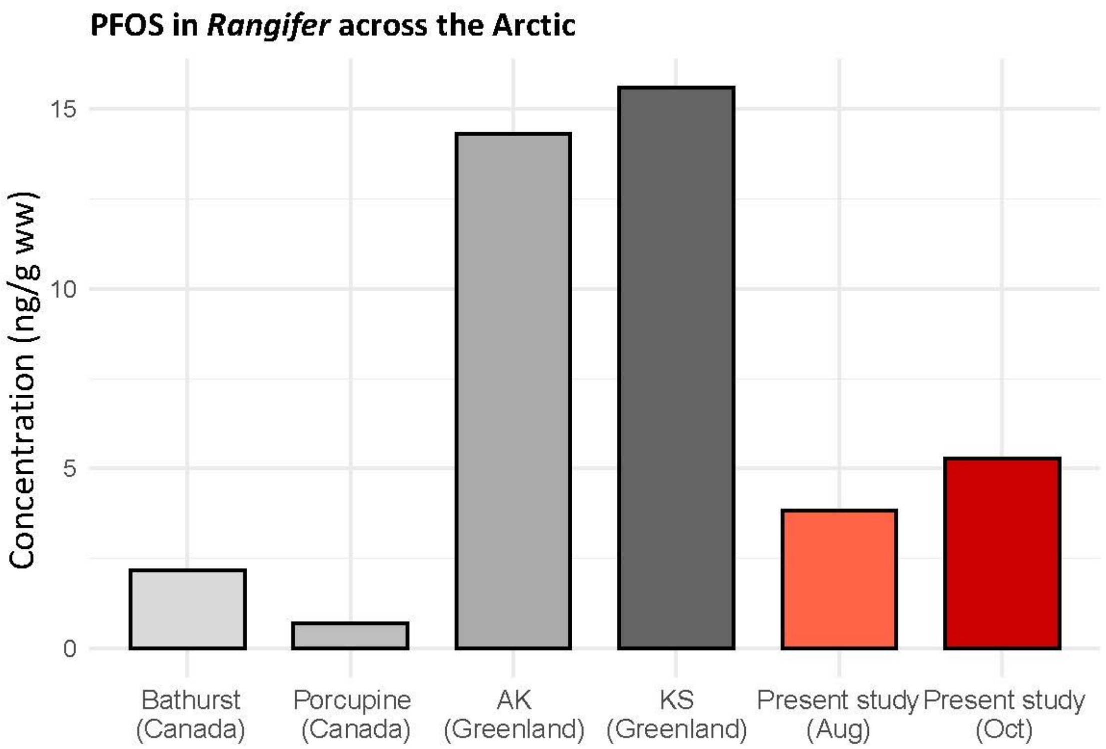

Figure S5. Hepatic PFOS concentrations (ww) from various Rangifer subspecies across the Arctic. Concentrations from Bathurst, Porcupine, Akia-Maniitsoq (AM) and Kangerlussuaq-Sisimiut (KS) are all reported in Roos et al. (2021) and are here visualised in different shades of grey. Average concentrations from both animals culled in August and October in the present study are visualised in shades of red.

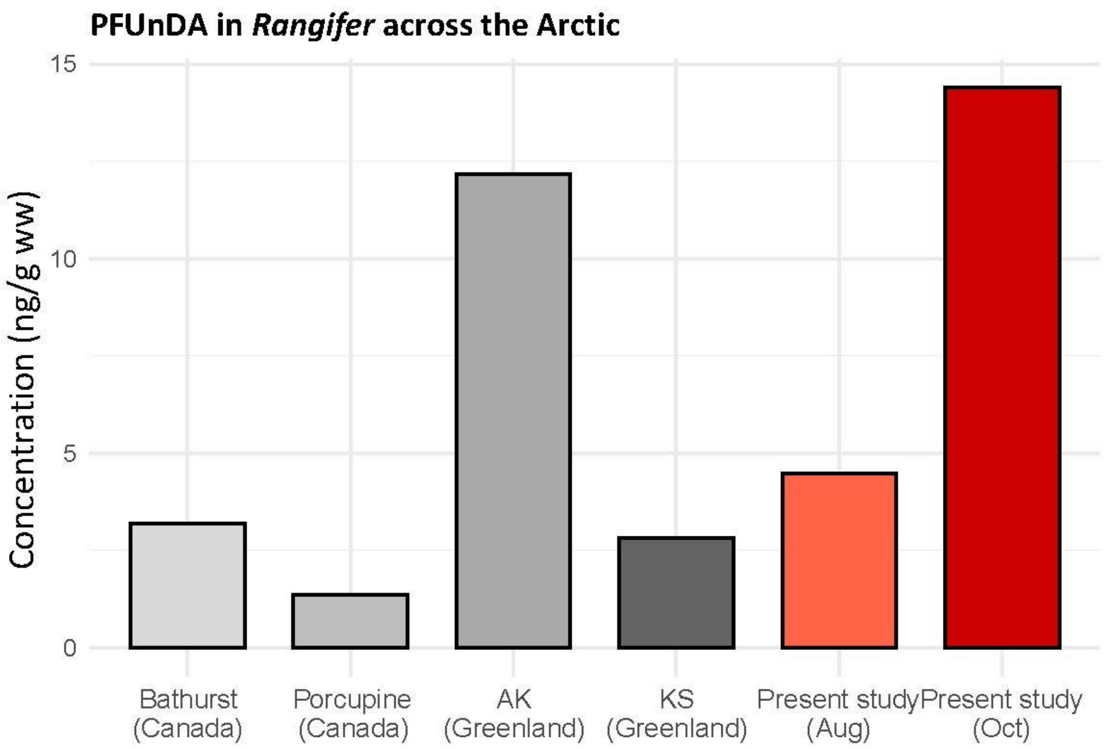

Figure S6. Hepatic PFuNDA concentrations (ww) from various Rangifer subspecies across the Arctic. Concentrations from Bathurst, Porcupine, Akia-Maniitsoq (AK) and Kangerlussuaq-Sisimiut (KS) are all reported in Roos et al. (2021) and are here visualised in different shades of grey. Average concentrations from both animals culled in August and October in the present study are visualised in shades of red.

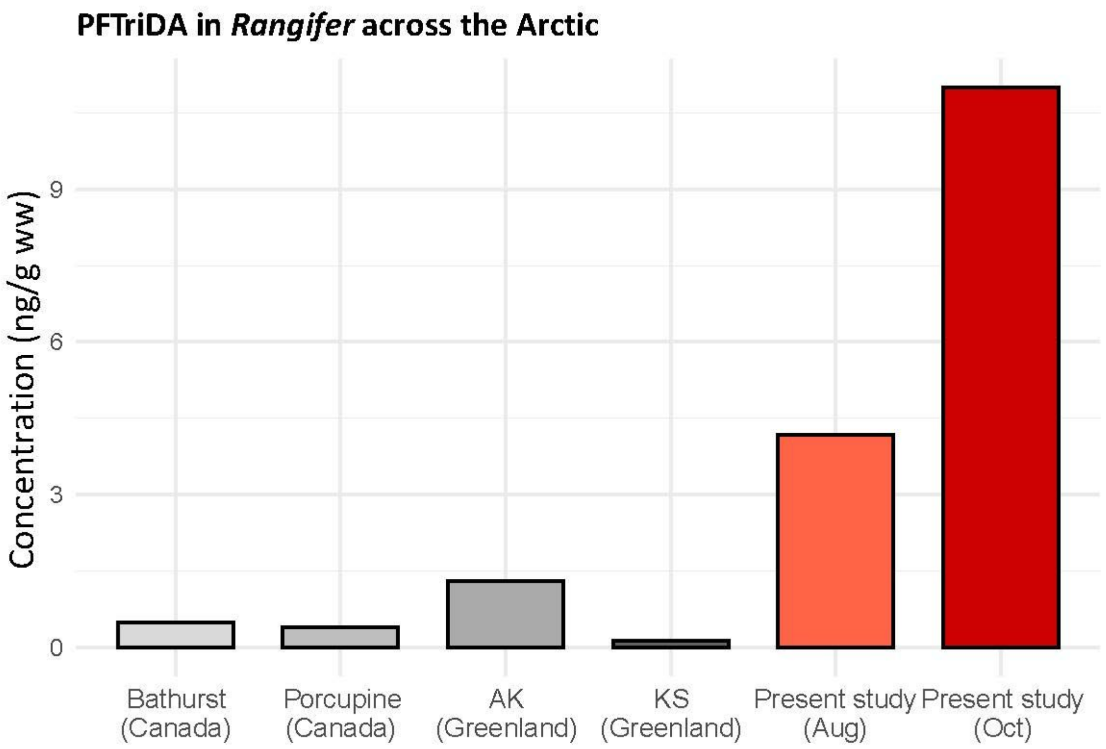

Figure S7. Hepatic PFTriDA concentrations (ww) from various *Rangifer* subspecies across the Arctic. Concentrations from Bathurst, Porcupine, Akia-Maniitsoq (AK) and Kangerlussuaq-Sisimiut (KS) are all reported in Roos et al. (2021) and are here visualised in different shades of grey. Average concentrations from both animals culled in August and October in the present study are visualised in shades of red.

REFERENCES

Ali Hassan, A., Rylander, C., Brustad, M., Sandanger, TorkjelM., 2012. Level of selected toxic elements in meat, liver, tallow and bone marrow of young semi-domesticated reindeer (*Rangifer tarandus tarandus L.*) from Northern Norway. International Journal of Circumpolar Health 71, 18187. https://doi.org/10.3402/ijch.v71i0.18187

Gamberg, M., Cuyler, C., Wang, X., 2016. Contaminants in two West Greenland caribou populations. Sci. Total Environ. 554–555, 329–336. https://doi.org/10.1016/j.scitotenv.2016.02.154

Institute of Nuclear Chemistry and Technology, 2010. Oriental Basma Tobacco Leaves (INCT-OBTL-5): Polish certified reference material for multielement trace analysis.

Institute of Nuclear Chemistry and Technology, & Gdańsk University of Technology., (n.d.). MODAS-3 Herring Tissue (M-3 HerTis): Polish certified reference material for multielement trace analysis with information values for selected radionuclides and some PCBs and PAHs.

Makarov, D.A., Ovcharenko, V.V., Nebera, E.A., Kozhushkevich, A.I., Shelepchikov, A.A., Turbabina, K.A., Kalantaenko, A.M., Bardyugov, N.S., Gergel, M.A., 2022. Geographical distribution of dioxins, cadmium, and mercury concentrations in reindeer liver, kidneys, and muscle in the Russian Far North. Environ. Sci. Pollut. Res. 29, 12176–12187. https://doi.org/10.1007/s11356-021-16310-2

O'Hara, T.M., George, J.C., Blake, J., Burek, K., Carroll, G., Dau, J., Bennett, L., McCoy, C.P., Gerard, P., Woshner, V., 2003. Investigation of Heavy Metals in a Large Mortality Event in Caribou of Northern Alaska. ARCTIC 56, 125–135. https://doi.org/10.14430/arctic608

Roos, A.M., Gamberg, M., Muir, D., Kärrman, A., Carlsson, P., Cuyler, C., Lind, Y., Bossi, R., Rigét, F., 2021. Perfluoroalkyl substances in circum-ArcticRangifer: caribou and reindeer. Environ. Sci. Pollut. Res. https://doi.org/10.1007/s11356-021-16729-7

Sait, S.T.L., Rinø, S.F., Gonzalez, S.V., Pastukhov, M.V., Poletaeva, V.I., Farkas, J., Jenssen, B.M., Ciesielski, T.M., Asimakopoulos, A.G., 2023. Occurrence and tissue distribution of 33 legacy and novel per- and polyfluoroalkyl substances (PFASs) in Baikal seals (Phoca sibirica). Sci. Total Environ. 889, 164096. https://doi.org/10.1016/j.scitotenv.2023.164096

Schuster, R.C., Gamberg, M., Dickson, C., Chan, H.M., 2011. Assessing risk of mercury exposure and nutritional benefits of consumption of caribou (Rangifer tarandus) in the Vuntut Gwitchin First Nation community of Old Crow, Yukon, Canada. Environ. Res. 111, 881–887. https://doi.org/10.1016/j.envres.2011.05.025
